# Long-Term Efficacy and Safety of Botulinum Toxin Injections in Dystonia

**DOI:** 10.3390/toxins5020249

**Published:** 2013-02-04

**Authors:** Juan Ramirez-Castaneda, Joseph Jankovic

**Affiliations:** Parkinson’s Disease Center and Movement Disorders Clinic, Department of Neurology, Baylor College of Medicine, Houston, TX 77030, USA; E-Mail: jlcastan@bcm.edu

**Keywords:** long-term, efficacy, safety, botulinum toxin, dystonia

## Abstract

Local chemodenervation with botulinum toxin (BoNT) injections to relax abnormally contracting muscles has been shown to be an effective and well-tolerated treatment in a variety of movement disorders and other neurological and non-neurological disorders. Despite almost 30 years of therapeutic use, there are only few studies of patients treated with BoNT injections over long period of time. These published data clearly support the conclusion that BoNT not only provides safe and effective symptomatic relief of dystonia but also long-term benefit and possibly even favorably modifying the natural history of this disease. The adverse events associated with chronic, periodic exposure to BoNT injections are generally minor and self-limiting. With the chronic use of BoNT and an expanding list of therapeutic indications, there is a need to carefully examine the existing data on the long-term efficacy and safety of BoNT. In this review we will highlight some of the aspects of long-term effects of BoNT, including efficacy, safety, and immunogenicity.

## 1. Introduction

BoNT is the most potent biologic toxin found in nature. Its pharmacological action results in inhibition of acetylcholine release into the neuromuscular junction. As a result of local chemodenervation BoNT relaxes abnormally contracting muscles which has been shown to be an effective treatment in a variety of movement disorders and other neurological and non-neurological disorders. After several months of deliberation and a requirement for various precautions, including the presence of resuscitation crash cart, the Baylor College of Medicine Institutional Review Board for Human Research first granted approval for the use of BoNT to one of the authors (JJ) who injected the first patient, a 45 year old woman with blepharospasm, on 12/15/1981. 

Since the first double-blind, placebo-controlled trial demonstrated efficacy of BoNT-A in cervical dystonia, published in 1986 [1], and in cranial-cervical dystonia, published in 1987 [2], BoNT has been used to treat hundreds of different conditions not only in Neurology but nearly all fields of Medicine. The results from the initial trial in cranial-cervical dystonia at Baylor College of Medicine [2], blepharospasm, a form of focal dystonia, was the first indication approved by the United States Food and Drug Administration (FDA) for the treatment with BoNT-A in 1989 [3]. 

Currently there are four BoNT products commercially available in the US approved for various therapeutic and cosmetic indications with the following non-proprietary and brand names: OnabotulinumtoxinA (Botox^®^ [Type A]); IncobotulinumtoxinA (Xeomin^®^ [Type A]); AbobotulinumtoxinA (Dysport^®^ [Type A]); RimabotulinumtoxinB (Myobloc^®^ [Type B]) [4]. In addition to FDA-approved indications, there is an expanding list of off-label therapeutic uses [5,6,7,8,9]. In this review we will highlight some of the aspects of long-term effects of BoNT, including efficacy, adverse effects, and immunogenicity. 

## 2. Long-Term Experience and Efficacy

Because it would not be feasible or ethical to conduct long-term double-blind, placebo-controlled studies, data from published longitudinal studies are limited by lack of objective assessments and relatively small sample size. Only a few studies have been published regarding the long-term effects of BoNT therapy for the treatment of dystonia over five years or longer (Table 1). In 2005 we published longitudinal follow-up data on 45 patients who had received BoNT treatments continuously for at least 12 years at Baylor College of Medicine Movement Disorders Clinic [10]. Patients were followed for a mean of 32 visits and a mean of 16 years. The most frequent reasons for treatment were cervical dystonia (43%), cranial dystonia (26%), and blepharospasm (11%). Patients’ response was rated according to a 0–4 scale (0 no effect to 4 marked improvement in severity and function). Over time, there was no significant change in latency and total duration of response to treatment and the dose per visit, peak duration of response, global rating, and peak effect showed incremental improvement over time. One third of patients developed side effects after their initial visit compared with 22% at their most recent visit. The most common side effects were dysphagia and ptosis. Antibody testing by the mouse protection assay (MPA) was performed in 22 patients due to non-responsiveness; blocking antibodies were confirmed in 4 (18%) of these patients. Sixteen of the antibody-negative patients resumed responsiveness by dose adjustment, whereas 2 persisted as non-responders. These findings suggest that BoNT is an effective long-term treatment with adequate and persistent therapeutic response and mild side effect profile. The relatively high frequency of immunoresistance was due to the use of original OnabotulinumtoxinA, which had a relatively high protein load (see below). 

A retrospective analysis of the long term efficacy of BoNT-A over a 10-year period in 235 subjects with cervical dystonia, hemifacial spasm, blepharospasm, and other focal/segmental dystonia found that at 2 years the highest response rate of sustained benefit (defined as continued improvement of 50% or more from baseline) was in patients with hemifacial spasm (96%), followed by patients with blepharospasm (92%) and with cervical dystonia (68%) [11]; this was similar at 5 years. The BoNT doses used during the first year were compared with doses used during second to fifth year, and doses used after the fifth year. Minor side effects developed in 27% of the patients at any one time, occurring in 4.5% of treatment cycles. Patient satisfaction increased after 5 years of treatment with an average reported benefit of 75.8%. 

Another retrospective review of 20 patients with focal hand dystonia followed for 10 years, up to 16 years showed mild average benefit in 11 subjects with a trend towards larger benefit in women [12]. This cohort required a gradual increase in dose over time, patients received a higher mean dose at the end of the follow-up period compared to the initial treatment (50 *vs*. 25 U respectively, *p* < 0.00005), which could partly be explained by a relatively low initial dose. Focal hand dystonia has a lower overall response rate, with about 50% of patients receiving at least mild benefit compared to 80% for cervical dystonia [13] and over 90% for blepharospasm [14]. More than one third of musicians reported long-term benefit and improvement in their performance ability in a retrospective review [15] of 84 (74 men) subjects with focal task-specific dystonia by estimating their playing ability compared to their skill level prior to onset of dystonic symptoms before and during EMG-guided BoNT treatment and rated their cumulative treatment response using a self-rating scale. 

Post-hoc analysis of two prospective, long-term, open-label extension studies of AbobotulinumtoxinA in cervical dystonia [16] demonstrated that treatment was generally well tolerated with no major differences in the incidence of adverse events. In both studies, patients were randomized to receive a single treatment cycle of AbobotulinumtoxinA 500 U diluted in 1 mL preservative-free normal saline or placebo. A total of 137 subjects entered the first double-blind, placebo-controlled study [17] and 131 received at least one treatment cycle of AbobotulinumtoxinA. A total of 116 subjects entered the second double-blind, placebo-controlled study [18], 108 of whom entered the open-label study. Based on the analysis of the combined cohort of 239 patients who entered the two-extension, open-label, studies all treatment cycles resulted in improvements in mean Toronto Western Spasmodic Torticollis Rating Scale (TWSTRS) total scores 4 weeks post-injection. The mean baseline TWSTRS scores at the time of the first cycle were 44.5 ± 8.8 and 42.5 ± 10.2 for the first and second studies, respectively; and the mean TWISTRS score changes from baseline were −13.7 ± 10.9 and −16.2 ± 11.5 for the first and second studies, respectively. At the time of the third cycle, the baseline scores were 41.0 ± 10.7 and 32.75 ± 11.7 for the first and second studies, respectively; and the mean changes were −12.2 ± 10.3 and −10.8 ± 13.0 for the first and second studies, respectively. However, increasing the dose of AbobotulinumtoxinA above the initial 500 U dose was not observed to result in an incremental improvement in response as measured by the TWSTRS.

A survey of 155 patients with cervical dystonia treated with BoNT-A injections over a period of 6 years [19], 133 (86.6%) individuals completed the survey; 78% (104) continued and 22% (29) stopped therapy. Two thirds of the patients who continued BoNT-A treatments reported the injections always helped, whereas one quarter estimated one set of injections did not help. One third of those continuing treatment reported the first injection was most helpful, whereas another one third felt all injections were similarly effective. After an initial adjustment, BoNT-A dosages and frequency of treatment remained stable.

A report of efficacy of BoNT-A in 78 patients with idiopathic cervical dystonia treated for a median of 5.5 years (range 1.5–10 years), as evaluated by the patients themselves and their neurologists, showed a high degree of patient satisfaction [20]. The effect of BoNT treatment was scored on a seven-point scale (0 excellent to 6 worse), being excellent, good or moderate in 85%. The independent scores of the treating neurologists were excellent, good or moderate in 92%, and correlated well with the patients’ scores. There was also a marked reduction of ‘Global Burden of Disease’, as expressed on the Visual Analog Scale (0 designating no burden and 10 the worse imaginable burden). By combining these outcome measures into two major groups, 67% of the patients were designated as having a good effect, and 33% had an unsatisfactory effect. 

There are currently several prospective observational studies that may provide useful information about the long-term efficacy and safety or BoNT treatment. These include (1) CD PROBE (cervical dystonia patient registry for observation of OnabotulinumtoxinA efficacy) [21]; (2) MOBILITY (prospective, non-randomized, observational, multi-center evaluation of health utility in patients receiving BOTOX^®^ for therapeutic use) [22]; (3) XCiDaBLE (an observational, prospective trial evaluating Xeomin^®^ (IncobotulinumtoxinA) for cervical dystonia or blepharospasm) [23]; and (4) ANCHOR-CD (a prospective, open-label, observational study of adult idiopathic cervical dystonia patients treated with AbobotulinumtoxinA) [24]. 

The published data provides evidence that BoNT products not only exert safe and effective symptomatic relief but also long-term benefits, favorably modifying the natural history of this disease, such as lower risk of contractures in patients with cervical dystonia [25]. The adverse events associated with chronic, periodic exposure to BoNT type A or B injections are generally minor and self-limiting, and even decrease over time [26]. The presence of neutralizing antibodies against the toxin is only responsible for a minor proportion of the small group of dystonia patients that develop persistent therapeutic resistance. 

The following Table summarizes the most relevant findings from published studies of efficacy, safety, and side effect profile of long-term BoNT therapy. The included studies were selected based on the main treating diagnosis of any type of dystonia with the periodic use of BoNT injections with a mean follow-up period of at least 5 years, including description of either total or per-session mean dose of the toxin, outcome measures of response/effectiveness, and report of the adverse events. If a study did not have at least a range follow-up period of 60 months, it was omitted from this table. 

**Table 1:** Long-term studies of movement disorders patients receiving BoNT injections including at least a 5 year follow-up or longer.

Author/Year; No. Pts.	Mean Follow-up Period	BoNT Indication; BoNT type	Mean Age (years)	Mean Dose (MU)	Response	Side Effects	Outcomes
Tan, *et al*., 1999 [27]; 162	4.4 ± 3.8 (range: 0.3 to 10 years)	Oromandibular dystonia (OMD); OnabotulinumtoxinA	57.9 ± 15.3	Per muscle: masseter 54.2 ± 15.2; submentalis 28.6 ± 16.7; Cumulative: 778.8 ± 884.4 (masseter) 226.1 ± 342 (submentalis)	68% had global rating ≥3; mean peak effect: 3.1 ± 1.0 [28]; mean global effect: 3.0 ± 1.0; mean total duration response: 16.4 ± 7.1 weeks	31% (most common: dysarthria and dysphagia), only 11% of all visits	Jaw-closing dystonia responds better than jaw-opening or mixed dystonias.
Kessler, *et al*., 1999 [29]; 303	3.2 (range: 1.3 to 5.9 years)	Cervical dystonia (CD); AbobotulinumtoxinA	41 (range: 17 to 69)	Per session: 778 ± 253; Median Cumulative Dose: 7430 (range: 2700 to 22,475)	Highly significant average score [30] reduction from 10 points on initial presentation to 4 points after 15 injections. The mean score decrease after the first injection was 3.7 ± 1.9 points [31]	75% (most common: dysphagia, neck muscle weakness, hoarseness, dry mouth), 22% of all visits	Most patients underwent dramatic improvement after the first few injections. However, the true quality of treatment cannot reliably be judged before the sixth to eighth injections. BoNT treatment is equally effective in pure rotational torticollis and in more complex forms of CD. The relative improvement was similar for all patients irrespective of their disease severity.
Hsiung, *et al*., 2002 [11]; 235	2–12 years	CD (45%), hemifacial spasm (HS) (30%), blepharospasm (BP) (15%), other focal or segmental dystonia (10%); Onabotulinumtoxin A	Range: 21–86 years; CD 48 (21–80); HS 57 (28–86); BP 60 (30–82)	CD 222, HS 29.4, BP 51.5	At 2 years, HS patients had the highest response rate of sustained benefit, with 96% reporting 50% or better improvement, followed by BP 92% and CD 68%, these sustained benefits were similar at 5 years	27% (most common: CD: dysphagia and muscle atrophy, HS: ptosis and facial asymmetry, BP: ptosis and dry eyes); 65% of adverse effects occurred during the first 4 years of treatment	Most of the patients showed a prolonged sustained benefit with repeat injections, with only slight decrease seen at 5 years compared with at 2 years. Most patients who responded had sustained benefit over 5 or more years. The highest frequency of side effects occurred in patient with BP, followed by patients with HS, and CD. The main reason for discontinuation of treatment was lack of benefit (primary or secondary resistance 39%).
Snir, *et al*., 2003 [32]; 27 [33]	BP: 33.5 ± 13.3 months (low dose) and 26.1 ± 11.0 months (high dose); HS: 23.8 ± 6.6 months (low dose) and 31.6 ± 8.6 months (high dose)	BP (17 patients) and HS (10 patients); Onabotulinumtoxin A	Men (14/27): 75.7 ± 9.2 and women (13/27): 74.0 ± 4.7	BP: 16.0 ± 1.4 (low dose) and 24.2 ± 1.4 (high dose); HS: 16.8 ± 1.2 (low dose) and 25.0 ± 1.8 (high dose)	BS: mean dose/patient changed from 16.0 ± 1.4 U (lower dose) to 24.2 ± 1.4 U (higher dose), the shift occurred after a mean of 8.8 ± 2.9 treatments per patient HS: mean dose/patient changed from 16.8 ± 1.2 U (lower dose) to 25.0± 1.8 U (higher dose), the shift occurred after a mean of 6.5± 2.3 treatments per patient	70% had dry eye, followed by ptosis and strabismus	The mean interval of relief was longer with the lower dose than with the higher dose in BS, and similar for both dose ranges in HS. The dose was increased over time by 50% to achieve 3 to 4 months of symptomatic relief with minimal complications. The HS group switched to the higher dose earlier than the BS group.
Haussermann, *et al*., 2004 [34]; 100	61.02 ± 54.53 months (median 49: range 3–143)	CD; AbobotulinumtoxinA	47.23 ± 14.28	Per session: 800.79 ± 241; Cumulative: 10,154.45 ± 10,202.96	Global subjective BoNT effect score [35] over the whole treatment period was 1.93 ± 1.18; at therapy onset Tsui severity scale: 8.98 ± 3.66; segmental or multisegmental spread of dystonia developed in 33% of patients during follow-up	34% (most common: weakness of cervical muscles, mild dysphagia, generalized weakness); 33% stopped therapy due to travel inconvenience and side effects	Patients showed high adherence to BoNT treatment over time. More than 60% of patients continued with BoNT injections after up to 12 years. The mean BoNTdose of 800 MU may have been a relatively high dose compared to other studies, increasing frequency of side effects.
Skogseid, *et al*., 2005 [20]; 78	5.5 (range: 1.5 to 10 years)	CD; OnabotulinumtoxinA	Range: 18–75	111 (range: 82 to 190)	The median VAS score of Global Burden of Disease [36] prior to treatment was 8.0 (4.8–10) and at the time of treatment evaluation it was 4.0 (1.0–8.0). The median difference in VAS scores prior to and at treatment evaluation in individual patients was 4.0 (1.0–7.0) *p* < 0.001. Median TWSTRS total score at the time of treatment evaluation was 33 in the total population, 31 in the ‘Good effect’ group and 37 in the “Unsatisfactory effect” group, *p* = 0.021	42% (most common: dysphagia, injection site pain, neck muscle weakness, hypophonia)	Longitudinal studies of individual patients showed that changes in the complexity of CD during treatment occur in 36%; 19% had developed less complex patterns and 17% more complex patterns. High degree of patient satisfaction with long-term BoNT treatment was confirmed by the patients’ effect scores being excellent, good or moderate in 85%, and by a marked reduction of “Global Burden of Disease” during treatment in most patients. The doctors’ independent effect scores were excellent, good or moderate in >90% of the patients, and correlated well with the patients’ scores.
	Mejia, *et al*., 2005 [10]; 45	15.8 ± 1.5 years	CD 37%, craniocervical dystonia 21%, cranial dystonia 13%, BP 9%, OMD 4%, FHD (focal hand dystonia) 4%, others [37]; BoNT-A and BoNT-B	51.8 ± 11.6	First session: 154.3 ± 98.9; most recent session: 221.2 ± 129.4; difference was statistically significant (*p* < 0.0001)	Average peak effect of 2.9 ± 1.5 (first injection) *vs*. 3.7 ± 0.6 (most recent injection); total duration response of 11.6 ± 7.1 weeks (first injection) *vs*. 15.4 ± 3.4 weeks (most recent injection)	Initial visit: 35% (most common: dysphagia and ptosis). Most recent injection visit: 22% (most common: ptosis and dysphagia)
	Schuele, *et al*., 2005 [15]; 84	23 months (range 2 to 76 months)	FHD; AbobotulinumtoxinA	45.9	Initial total dose per treatment: 126.9 (range: 5 to 420); last total dose per treatment: 112.2 (range; 3 to 1000)	58 (69%) of the musicians experienced benefit, among them 38 indicated that the treatment led to noticeable improvement in their performance ability	98% patients: weakness; 56% reported excessive weakness after at least one injection preceding the period of max improvement
Berman, *et al*., 2005 [38]; 24 [39]	26.2 ± 20.4 months (range: 3 to 64 months)	CD; BoNT-B	60.4 ± 12.0 (range: 36 to 82)	14,828 ± 6824 (range: 2500 to 28,000)	Many patients required dose escalation to obtain an optimal response. In the last visit 12 patients demonstrated ongoing benefit and 50% became nonresponders (33% primary and 66% secondary)	Out of 87 injections with evaluable data: 23% had minor adverse events: dry mouth,neck pain, dysphagia, and headache	The use of BoNT-B at doses greater than the current recommended guidelines may be necessary to adequately treat some patients with severe CD both with and without prior BoNT-A resistance. The majority but not all BoNT-A resistant CD patients treated with BoNT-B became resistant to BoNT-B within 2 years or 5 injection cycles.
Mohammadi, *et al*., 2009 [40]; 207	AbobotulinumtoxinA: 7.3 ± 3.1 years (max 14 years); OnabotulinumtoxinA: 5.0 ± 2.2 (max 12 years)	CD; AbobotulinumtoxinA 163, OnabotulinumtoxinA 44	58 ± 27 (range: 22–95)	AbobotulinumtoxinA: 389 ± 144; OnabotulinumtoxinA: 145 ± 44	The duration of treatment effect was 11 ± 1.6 weeks in the AbobotulinumtoxinA group and 10 ± 2.4 in the OnabotulinumtoxinA group. The GGI [41] was rated 2.5 ± 0.3 for AbobotulinumtoxinA and 2.2 ± 0.4 for OnabotulinumtoxinA	Neck muscle weakness in 5% and 7% of treatment sessions, dysphagia in 8% and 9% and pain at injection site in 9% and 6% for AbobotulinumtoxinA and OnabotulinumtoxinA, respectively	Satisfying effects with relatively low mean doses of AbobotulinumtoxinA and low to average mean doses of OnabotulinumtoxinA. Lower rates of side effects without difference between AbobotulinumtoxinA and OnabotulimtoxinA. Weakness of the study may be the fact that clinical score scales such as the Tsui score or the TWSTRS were not used. However the GGI proved to be useful as a practical alternative.
Bentivoglio, *et al*., 2009 [42]; 128	15-year period	BP; OnabotulinumtoxinA and AbobotulinumtoxinA [43]	At onset: 57.7 ± 10.3 (range: 6 to 81)	34 ± 15 (range: 7.5 to 140) for OnabotulinumtoxinA; 152 ± 54 (range: 40 to 400) for AbobotulinumtoxinA	Mean duration of clinical improvement was higher after the injection of AbobotulinumtoxinA (80.1 ± 36.3 days) than OnabotulinumtoxinA (66.2 ± 39.8 days) with *p* < 0.05. In a six-point scale [44], the mean efficacy of both treatments was 3.60 ± 1.3 (3.51 ± 1.4 OnabotulinumtoxinA and 3.85 ± 1.2 AbobotulinumtoxinA, *p* < 0.01)	21.8% of OnabotulinumtoxinA and 31.6% of AbobotulinumtoxinA patients; most common: palpebral ptosis in both followed by hematoma after OnabotulinumtoxinA and diplopia after AbobotulinumtoxinA	The dose of both BoNTs were significantly increased over time. The differences in outcomes and side effects suggest that, albeit the active drug is the same, OnabotulinumtoxinA and AbobotulinumtoxinA should be considered as two different drugs. No correlation was found between dose and occurrence of side effects. Treatment failure (less that 20% of amelioration) occurred in 7.7% of OnabotulinumtoxinA treatments and 3.6% of AbobotulinumtoxinA, *p* = 0.0093).
Cillino, *et al*., 2010 [14], 155 [45]	Followed up for at least 10 years	BP: 73 patients, HS: 58 patients, and spastic entropion (SE): 24 patients; OnabotulinumtoxinA	BP: 71.4 ± 12.3; HS: 71.7 ± 11.4; SE: 78.6 ± 8.9 (*p* = 0.024)	BP: 28.2 ± 12.2; HS: 18.7 ± 9.4; SE: 10.6 ± 4.7	Mean effect duration for patient in weeks: BP: 18.2 ± 12.3; HS: 20.6 ± 11.6; SE: 13.7 ± 7.0 (inter-group difference: *p* = 0.009). Significant intra-group differences were found for mean dosages, which increased significantly (*p* < 0.05) after the first 3 or 4 doses of treatment for both BP and HS groups	31.5% in BP, 31.0% in HS, and 4.2% in SE. Most common: upper lid ptosis, diplopia, ecchymosis, and injection-site bruising. Total side effects difference among 3 groups: *p* = 0.023	There was statistically significant difference in the mean BoNT-A dose received by patients among the 3 groups (*p* < 0.0005). A total of 96% of patients with BP, 98% of patient with HS, and 100% of patients with SE had significant relief of their symptoms. Subgroup analysis according to age (< or ≥ 65 years) indicates a significant increase in duration of relief and mean doses over the follow-up period in older but not younger BP and HS patients.
Lungu, *et al*., 2011 [12]; 20 [46]	13.6 ± 2.5 years	FHD; Ona botulinumtoxinA (except for one single injection of RimabotulinumtoxinB in one patient)	46.6 ± 9.45	Per session: 46.4 ± 24.6	Most patients (11 of 20) experienced mild average benefit (grade 2) [47]; patients received a higher mean dose at the end of the follow-up period compared to the initial treatment (50 *vs*.25 MU respectively, *p* < 0.00005). The benefit was higher with the last injection compared to the initial (47% *vs*. 26%, *p* = 0.039)	All patients tolerated the discomfort of multiple injections well; there were no serious adverse effects; two patients discontinued treatment due to insufficient response	There was large variability in the frequency of treatments, likely reflecting the fact that while FHD makes particular activities difficult or impossible, it is not otherwise disabling or painful. The musicians were more likely to wait longer between injections (19.9 ± 12.4 months for musicians *vs*. 7.7 ± 2.3 for nonmusicians, *p* < 0.002). Patients continued therapy for over 10 years in spite of only mild benefit, suggesting that even partial improvement may be worthwhile.
Ramirez-Castaneda and Jankovic, 2012; [48] 104	19.4 ± 2.9 years	Dystonia; BoNT-A and BoNT-B	48.9 ± 12.4	Per session (at time of last injection): 300 ± 205	70% had global rating ≥3; mean peak effect: 3.7 ± 0.45; mean duration of maximal response: 15.5 ± 7.0 weeks.	13.4% at the time of their last visit; most common: blurred vision and dysphagia	Persistent improvement of dystonia severity and function is maintained over time with minimal adverse effects, thus supporting the conclusion that BoNT is a safe and effective long-term treatment for focal and segmental dystonia.

## 3. Immunogenicity and Therapeutic Response

Lack of response to BoNT treatment for dystonia and other movement disorders may occur for myriad reasons including inadequate dose, inappropriate muscle selection, concomitant drug therapy, dynamic disease change, and the development of neutralizing antibodies [10,49,50]. As a foreign protein, BoNT is inherently immunogenic, but because it is administered in only extremely small quantities at relatively long inter-dose intervals, most patients do not develop immunoresistance to BoNT. Nevertheless, BoNT elicits antibodies in some patients that can neutralize the neurotoxin, as a result of which the patient becomes unresponsive that type of BoNT [5]. The presence of neutralizing antibodies that act by blocking binding of the BoNT complex to the presynaptic membrane is one explanation for the occasional reports of lack of response to BoNT injections [51]. 

Although immunogenicity was an important consideration in the past [52], in today’s clinical practice poor or no response to BoNT is more frequently due to factors other than immunogenicity [29,49,53,54]. A prospective, open-label, multicenter study of 326 patients with cervical dystonia evaluated the immunogenicity of BoNT-A [49]. Seventy-seven percent subjects completed the study, receiving a mean dose per session ranging from 148.4 to 213.0 U and an overall mean cumulative dose of 1576 U over a mean of 2.5 years. The two main reasons for discontinuation of BoNT treatment were loss to follow-up (28%) and adverse events (17%). Four of 326 (1.2%) subjects developed neutralizing antibodies as measured by the MPA, one continued to respond to treatments, and the other three lost clinical responsiveness. Two of the 3 patients had a clinical resistance test (test injection of 20 U of BoNT-A placed unilaterally into the frontalis) performed and only one was found to indicate unresponsiveness. Although the MPA is considered the “gold standard” with relatively high specificity, it may lack sensitivity in identifying those with low antibody titers, thus underestimating the number of subjects with immunoresistance [55].

The previous presence of neutralizing antibodies may not be a predictor of response to BoNT therapy. A randomized, multicenter, double-blind study evaluated the efficacy of OnabotulinumtoxinA *versus* placebo for the treatment of cervical dystonia. The impact of the subjects’ neutralizing antibody status at study entry on clinical response was analyzed. A total of 214 subjects were enrolled in period 1 and received treatment with OnabotulinumtoxinA at a mean dose of 241.2 U (range, 95–360 U). One hundred seventy subjects entered period 2 and were randomized to receive Onabotulinumtoxin A (*n* = 88) or placebo (*n* = 82). Of the 114 subjects with valid samples at both study entry and exit, 19 subjects showed the presence of neutralizing antibodies at study exit; however, 17 of these samples tested positive at study entry, indicating that only 2 (2%) of 97 subjects seroconverted from negative to positive over the course of 2 treatments in this 22- to 26-week study. The two subjects remained responsive to OnabotulinumtoxinA during both the open and blinded treatment periods. OnabotulinumtoxinA improved disabling features associated with cervical dystonia, with a robust response in both groups compared to baseline [53].

BoNT complexing proteins elicit antibodies in about half of patients [56,57], but only a small proportion of induced antibodies are actually neutralizing (blocking) antibodies. Furthermore, there is evidence suggesting that the anamnestic immunologic response to BoNT can wane with time, but can be reactivated by repeat exposure to the same type of BoNT. BoNT positive antibody status can revert to negative status over time. Seven patients, with a mean age 56 years, six with cervical dystonia and one with oromandibular dystonia, immunoresistant to BoNT-A therapy with positive antibodies, were retested by MPA for the persistence of neutralizing antibodies [58]. The mean duration of symptoms was 197 months and received a total mean cumulative dose of 1659 U. All patients reverted from BoNT-A positive antibody status to negative. Six antibody positive patients were reinjected with BoNT-A after they reverted to negative antibody status. All improved after the reinjections, but three lost their response to subsequent injections, which was confirmed by re-emergence of antibodies. In four of these patients, response to repeat BoNT-A injections was confirmed by weakness of the right frontalis muscle injected simultaneously. This unique group of patients responded favorably to repeat BoNT-A injections, but some lost the benefit with subsequent injections. 

Differences among BoNT preparations, both within and between serotypes, lead to differences in clinical performance, safety, antigenicity, and specificity [59]. Although recently challenged [54], RimabotulinumtoxinB (Myobloc^®^ [Type B]) has been reported to have a high rate of immunogenicity [60,61]. This high frequency of antibody formation may be possibly related to a large amount of protein in the BoNT-B product. IncobotulinumtoxinA (Xeomin^®^ [Type A]); is a preparation of BoNT type A that is structurally free of NAPs with high specific biological activity [62], but it is not yet clear whether this property translates into lower antigenicity [63]. 

## 4. Adverse Effects and Treatment Failures

Despite the widespread commercial acceptance of BoNT for cervical dystonia, there is lack of adequate follow-up data to determine the reasons why patients stop therapy. In a survey of 155 patients with cervical dystonia who were treated over 6 years with BoNT of 133 (86.6%) individuals who returned the surveys, 104 continued and 29 stopped therapy. From the latter group, 11 individuals had only received one or two injections. Prior surgical treatment for cervical dystonia did not influence their decision to stop therapy [19].

Weakness in cervical musculature and dysphagia are the most common BoNT treatment-related side effects in patients with CD [19,29,64]. Dropout from treatment can also be secondary to symptom improvement, remission, or location/access-related issues [11,29,34]. In one study involving a total of 616 patients [29], 155 of whom were lost to follow-up, there was a total of 173 reasons for discontinuation. A large group of those patients (48) had not actually discontinued BoNT-A therapy but had merely switched to other treatment centers. The corrected overall dropout rate was thus 20% (126/616 patients). The common reasons (number of patients/percentage) for withdrawing were primary nonresponse (33/26.1%), secondary nonresponse (17/13.4%), adverse events (27/21.4%), remission/improvement (26/20.6%), and other (34/26.9%) which included inconvenience of cost, travel, relocation, discontinuation by physician, lost to follow-up, and unknown. 

In a similar 10-year retrospective analysis of 235 clinic patients with various movement disorders treated with BoNT-A, 28% of patients discontinued treatment during the follow-up period due to a variety of reasons [11]. Overall, 9.1% (21 patients) had primary resistance, which is highly surprising as in our practice we almost never see primary nonresponders unless they were previously vaccinated against BoNT, as is the case in some military personnel. Apparently, 7.5% 18 patients) developed secondary resistance, but it is not clear who this was verified and no MPA or other assays were apparently performed. Only 1.3% of patients discontinued therapy due to intolerable adverse effects; discontinuation for any reason was also low after 5 years. The main reason for stopping treatment was lack of benefit which may be secondary to inadequate muscle selection or injection technique, evolution of primary disease, or development of neutralizing antibodies. Another long-term cohort study of 100 cervical dystonia patients treated with AbobotulinumtoxinA followed for an average of 5 years [34] showed high adherence to BoNT treatment over time. More than 60% (57 of 90) of these patients continued with BoNT injections after up to 12 years. The most common reasons for stopping treatment were inconvenience (long travel distances, costs) 36% (12 of 33), side effects 33% (11 of 33), and significant improvement of dystonia 15% (5 of 33). Other reasons for discontinuation were secondary nonresponse (3), primary nonresponse (1), and complete remission (1). 

## 5. Conclusions and Future Directions

This review of longitudinal data provides evidence of long-term efficacy and safety of BoNT in the treatment of dystonia. In addition, chronic BoNT treatment has changed the natural history of dystonia. For example, contractures, which often occurred as a consequence of prolonged abnormal posture, are now rarely seen due to prevention of this dystonia-related complication by early institution of BoNT therapy. We chose to focus on dystonia as this is the earliest approved indication [2,65], but similar longitudinal experience is accumulating in other clinical conditions chronically treated with BoNT. 

With the development of new BoNT preparations that seem to have lower risk for antigenicity, immunoresistance appears to be a less important cause of loss of response than in the past. Further longitudinal data are needed to determine which rating scales and quality of life outcome measures are best suited for long-term follow-up studies and to better understand the reasons for withdrawal from continued BoNT therapy. 

Relative advantages and disadvantages of the various BoNT products need to be addressed not only in short-term, controlled studies but in well-designed long-term, observational studies. Although currently available BoNT drugs are safe and effective, the research and development of new products should focus not only on issues related with antigenicity, but also on selectivity based on duration of action, diffusion properties, and novel delivery routes. Future studies should also evaluate the most appropriate method of muscle selection for BoNT injection and techniques to prolong its effects. 

Finally, there is a need to study the long-term effects of BoNT on central neurophysiologic changes and neuroplasticity. The notion of central remodeling of cortical motor maps as a result of reduced afferent input following BoNT [66] needs to be further explored as a potential neurophysiologic and clinical consequence of long-term BoNT therapy.

## References

[1] Tsui J., Eisen A., Stoessl A., Calne D. (1986). Double-blind study of botulinum toxin in spasmodic torticollis. Lancet.

[2] Jankovic J., Orman J. (1987). Botulinum A toxin for cranial-cervical dystonia. Neurology.

[3] Costa J., Espirito-Santo C.C., Borger A.A., Ferreira J., Coelho M.M., Moore P., Sampaio C. (2009). Botulinum toxin type A therapy for blepharospasm (Review). Cochrane Database Syst. Rev..

[4] U.S. Food and Drug Administration Drugs@FDA/FDA Approved Drug Products. http://www.accessdata.fda.gov/scripts/cder/drugsatfda/index.cfm.

[5] Jankovic J., Albanese A., Atassi M.Z., Dolly J.O., Hallett M., Mayer N.H. (2009). Botulinum Toxin: Therapeutic Clinical Practice and Science.

[6] Jankovic J. (2009). Disease-oriented approach to botulinum toxin use. Toxicon.

[7] Jankovic J., Kompoliti K., Verhagen L. (2010). Botulinum Toxin. Encyclopedia of Movement Disorders.

[8] Mehanna R., Jankovic J. (2012). Botulinum Neurotoxins as Therapeutics. Handbook of Neurotoxicity.

[9] Thenganatt M., Fahn S. (2012). Botulinum toxin for the treatment of movement disorders. Curr. Neurol. Neurosci. Rep..

[10] Mejia N., Vuong K., Jankovic J. (2005). Long-term botulinum toxin efficacy, safety, and immunogenicity. Mov. Disord..

[11] Hsiung G.-Y.R., Das S.K., Ranawaya R., Lafontaine A.-L., Suchowersky O. (2002). Long-term efficacy of botulinum toxin A in treatment of various movement disorders over a 10-year period. Mov. Disord..

[12] Lungu C., Karp B., Alter K., Zolbrod R., Hallett M. (2011). Long-term follow-up of botulinum toxin therapy for focal hand dystonia: Outcome at 10 years or more. Mov. Disord..

[13] Jankovic J. (2006). Botulinum toxin therapy for cevical dystonia. Neurotox. Res..

[14] Cillino S., Raimondi G., Guepratte N., Damaiani S., Cillino M., di Pace F., Casuccio A. (2010). Long-term efficacy of botulinum toxin A for treatment of blepharospasm, hemifacial spasm, and spastic entropion: A multicentre study using two drug-dose escalation indexes. Eye.

[15] Schuele S., Jabusch H.C., Lederman R.J., Altenmüller E. (2005). Botulinum toxin injections in the treatment of musician’s dystonia. Neurology.

[16] Hauser R.A., Truong D., Hubble J., Coleman C., Beffy J.L., Chang S., Picaut P. (2012). Abobotulinumtoxin A (Dysport) dosing in cervical dystonia: An exploratory analysis of two large open-label extension studies. J. Neural Transm..

[17] Truong D., Duane D.D., Jankovic J., Singer C., Seeberger L.C., Comella C.L., Lew M.F., Rodnitzky R.L., Danisi F.O., Sutton J.P. (2005). Efficacy and safety of botulinum type A toxin (dysport) in cervical dystonia: Results of the first US randomized, double-blind, placebo-controlled study. Mov. Disord..

[18] Truong D., Matthew B., Lew M., Brashear A., Jankovic J., Molho E., Orlova O., Timerbaeva S. (2010). Global dysport cervical dystonia study group. Long-term efficacy and safety of botulinum toxin type A (dysport) in cervical dystonia. Parkinsonism Relat. Disord..

[19] Brashear A., Bergan K., Wojcieszek J., Siemers E.R., Ambrosius W. (2000). Patients’ perception of stopping or continuing treatment of cervical dystonia with botulinum toxin Type A. Mov. Disord..

[20] Skogseid I., Kerty E. (2005). The course of cervical dystonia and patient satisfaction with long-term botulinum toxin A treatment. Eur. J. Neurol..

[21] Jankovic J., Adler C.H., Charles P.D., Comella C., Stacy M., Schwartz M., Sutch S.M., Brin M.F., Papapetropoulos S. (2011). Rationale and design of a prospective study: Cervical dystonia patient registry for observation of onabotulinumtoxin A efficacy (CD PROBE). BMC Neurol..

[22] ClinicalTrials.gov. ClinicalTrials.gov Identifier: NCT00535938. NCT00535938.

[23] Jankovic J., Thomas M., Vasquez A., Sethi K., Verma A., Pappert E.J., Fernandez H.H. (2012). XCiDaBLE: A phase 4, observational, prospective trial evaluating incobotulinum toxin A for cervical dystonia (CD) or blepharospasm in the United States preliminary baseline results on the health impact of CD on patients using the cervical dystonia impact profile. Mov. Disord..

[24] Trosch R., LeWitt P., Lew M., Adler C., Clary C., Silay Y., Marchese D., Comella C. ANCHOR-CD. Abobotulinum Toxin A Neurotoxin: Clinical & Health Economics Outcomes Registry in Cervical Dystonia): A Multicenter, Observational Study of Dysport in Cervical Dystonia: Baseline Data and Interim Outcomes Data. Proceedings of 64th AAN Annual Meeting.

[25] Jankovic J. (2004). Botulinum toxin in clinical practice. J. Neurol. Neurosurg. Psychiatry.

[26] Colosimo C., Tiple D., Berardelli A. (2012). Efficacy and safety of long-term botulinum toxin treatment in craniocervical dystonia: A systematic review. Neurotox. Res..

[27] Tan E-K., Jankovic J. (1999). Botulinum toxin A in patients with oromandibular dystonia. Neurology.

[B28-toxins-05-00249] 28.0 to 4 “peak effect” scale (0: no effect; 1: mild effect, no functional improvement; 2: moderate improvement, no change in functional disability; 3: moderate change in severity and function; and 4: marked improvement in severity and function”).

[29] Kessler K., Skutta M., Benecke R. (1999). Long-term treatment of cervical dystonia with botulinum toxin A: Efficacy, safety, and antibody frequency. J. Neurol..

[B30-toxins-05-00249] 30.Severity of symptoms was rated according to a modified Tsui score [67]: 15-point score (intermittent *versus* constant head deviation in degrees (0°–15°, 15°–30°, >30°), head tremor (absent, slightly, moderate, severe), degree of involuntary shoulder elevation, level of pain in the neck/shoulder area).

[B31-toxins-05-00249] 31.The patients were divided into subgroups by their response to the first injection; all 3 groups had the same initial score: poor responders (*n* = 47), average responders (*n* = 208), and good responders (*n* = 48).

[32] Snir M., Weinberger D., Bourla D., Kristal-Shalit O., Dotan G., Axer-Siegel R. (2003). Quantitative changes in botulinum toxin A treatment over time in patients with essential blepharospasm and idiopathic hemifacial spasm. Am. J. Ophthalmol..

[B33-toxins-05-00249] 33.All patients initially received 12 or more courses of treatment with a lower dose (≤20 U) and were then switched to a higher dose (>20 U). The main outcome measures were the shift in the dose-response relationship between the lower and higher doses.

[34] Haussermann P., Marczoch S., Klinger C., Landgrebe M., Conrad B., Ceballos-Baumann A. (2004). Long-term follow-up of cervical dystonia patients treated with botulinum toxin A. Mov. Disord..

[B35-toxins-05-00249] 35.Subjective rating scale going from −4 (very bad effect in all sessions) to +4 (very good effect in all treatment sessions).

[B36-toxins-05-00249] 36.VAS: Visual analog scale, score 0–10, 0 designating no burden and 10 the worst imaginable burden.

[B37-toxins-05-00249] 37.Hemifacial spasm 2%, hemidystonia 2%, cranial dystonia and dysphonia 2%, craniocervical dystonia and dysphonia 2%, dysphonia 2%, and cervical dystonia and arm dystonia 2%.

[38] Berman B., Seeberger L., Kumar R. (2005). Long-term safety, efficacy, dosing, and development of resistance with botulinum toxin type B in cervical dystonia. Mov. Disord..

[B39-toxins-05-00249] 39.All 24 cervical dystonia patients had been previously treated with BoNT-A with a mean duration of treatment of 63.2 (5–168 months), and a mean dose with good response of 235.6 (150-300 units). There were 12 patients with definite, 7 with probable, and 1 with possible resistance. Only 4 patients demonstrated no evidence of resistance to BoNT-A.

[40] Mohammadi B., Buhr N., Bigalke H., Krampfl K., Dengler R., Kollewe K. (2009). A long-term follow-up of botulinum toxin A in cervical dystonia. Neurol. Res..

[B41-toxins-05-00249] 41.GGI: Global clinical improvement 0–3 scale (0, no effect; 1, slight; 2, moderate; and 3, marked improvement in severity and function).

[42] Bentivoglio A.R., Fasano A., Ialongo T., Soleti F., Lo Fermo S., Albanese A. (2009). Fifteen-year experience in treating blepharospasm with botox or dysport: Same toxin, two drugs. Neurotox. Res..

[B43-toxins-05-00249] 43.Total of 1341 treatments: Botox in 1009 and Dysport in 332 treatments.

[B44-toxins-05-00249] 44.Response was assessed by global rating from 0 to 6 (0, no effect; 1, effect less than 20%; 2, effect ranging between 20% and 40%; 3, effect ranging between 40% and 60%; 4, effect ranging between 60% and 80%; 5, effect ranging between 80% and 90%; 6, complete resolution of blepharospasm).

[B45-toxins-05-00249] 45.A total of 173 patients completed the 10-year follow-up: 83 patients with BP, 65 with HS, and 25 with SE. Of these, 18 did not attend follow-up visits for 12 months or more and were thus considered as “dropouts”.

[B46-toxins-05-00249] 46.Dystonia type: writing, 9; musician, 5 (piano, 2; guitar, 1; drums, 1; trumpet, 1); typing, 1; and mixed, 5.

[B47-toxins-05-00249] 47.Subjective scale based on percent restoration of normal function: 0 none, 1 minimal (1%–25% restoration of function), 2 mild (26%–50%), 3 moderate (51%–75%), 4 excellent (76%–100%).

[48] Ramirez-Castaneda J., Jankovic J. (2013). Long-term efficacy, safety, and side effect profile of botulinum toxin injections in dystonia. Neurology.

[49] Brin M.F., Comella C.L., Jankovic J., Lai F., Naumann M. (2008). CD-017 BoNTA Study Group. Long-term treatment with botulinum toxin type A in cervical dystonia has low immunogenicity by mouse protection assay. Mov. Disord..

[50] Oshima M., Deitiker P.R., Jankovic J., Duane D.D., Aoki K.R., Atassi M.Z. (2011). Human T-cell responses to botulinum neurotoxin proliferative responses *in vitro* of lymphocytes from botulinum neurotoxin A-treated movement disorder patients. J. Neuroimmunol..

[51] Jankovic J., Schwartz K. (1995). Response and immunoresistance to botulinum toxin injections. Neurology.

[52] Jankovic J., Vuong K., Ahsan J. (2003). Comparison of efficacy and immunogenicity of original *versus* current botulinum toxin in cervical dystonia. Neurology.

[53] Charles D., Brashear A., Hauser R.A., Li H.I., Boo L.M., Brin M.F., CD 140 Study Group (2012). Efficacy, tolerability, and immunogenicity of onabotulinum toxinA in a randomized double-blind placebo-controlled trial for cervical dystonia. Clin. Neuropharmacol..

[54] Chinnapongse R.B., Lew M.F., Ferreira J.J., Gullo K.L., Nemeth P.R., Zhang Y. (2012). Immunogenicitgy and long-term efficacy of botulinum toxin B in the treatment of cervical dystonia: Report of 4 prospective, multicenter trials. Clin. Neuropharmacol..

[55] Hatheway C.L., Dang C., Jankovic J., Hallett M. (1994). Immunogenicity of the Neurotoxins of Clostridium Botulinum. Therapy with Botulinum Toxin.

[56] Goschel H., Wohlfarth K., Frevert J., Dengler R., Bidalke H. (1997). Botulinum A toxin therapy: Neutralizing and nonneutralizing antibodies—Therapeutic consequences. Exp. Neurol..

[57] Critchfield J. (2002). Considering the immune response to botulinum toxin. Clin. J. Pain.

[58] Sankhla C., Jankovic J., Duane D. (1998). Variability of the immunologic and clinical response in dystonic patients immunoresistant to botulinum toxin injections. Mov. Disord..

[59] Aoki K. (2001). A Comparison of the safety margins of botulinum neurotoxin serotypes A, B, and F in mice. Toxicon.

[60] Dressler D., Bigalke H., Benecke R. (2003). Botulinum toxin type B in antibody-induced botulinum toxin type A therapy failure. J. Neurol..

[61] Jankovic J., Hunter C., Dolimbek B. (2006). Clinico-immunologic aspects of botulinum toxin type B treatment of cervical dystonia. Neurology.

[62] Jimenez-Shahed J. (2012). A new treatment for focal dystonias: Incobotulinum toxin A (Xeomin), a botulinum neurotoxin type A free from complexing proteins. Neuropsychiatr. Dis. Treat..

[63] Wohlfarth K., Muller C., Sassin I., Comes G., Grafe S. (2007). Neurophysiological double-blind trial of a botulinum neurotoxin type A free of complexing proteins. Clin. Neuropharmacol..

[64] Jankovic J. (2006). Treatment of dystonia. Lancet Neurol..

[65] Jankovic J., Schwartz K.S. (1993). Longitudinal experience with botulinum toxin injections for treatment of blepharospasm and cervical dystonia. Neurology.

[66] Kojovic M., Caronni A., Bologna M., Rothwell J.C., Bhatia K.P., Edwards M.J. (2011). Botulinum toxin injections reduce associative plasticity in patients with primary dystonia. Mov. Disord..

[67] Tsui J.K., Eisen A., Mak E., Carruthers J., Scott A., Calne D.B. (1985). A pilot study on the use of botulinum toxin in spasmodic torticollis. Can. J. Neurol. Sci..

